# The relationship between infectious agents and juvenile dermatomyositis: a narrative update from the pediatric perspective

**DOI:** 10.3389/fimmu.2024.1377952

**Published:** 2024-04-10

**Authors:** Chiara Sassetti, Claudia Borrelli, Martha Mazuy, Ida Turrini, Donato Rigante, Susanna Esposito

**Affiliations:** ^1^ Pediatric Clinic, University Hospital, Department of Medicine and Surgery, University of Parma, Parma, Italy; ^2^ Department of Child Neurology and Psychiatric Unit, Fondazione Policlinico Universitario A. Gemelli IRCCS, Rome, Italy; ^3^ Department of Life Sciences and Public Health, Fondazione Policlinico Universitario A. Gemelli IRCCS, Rome, Italy; ^4^ Università Cattolica Sacro Cuore, Rome, Italy

**Keywords:** juvenile dermatomyositis, pediatric infectious disease, autoimmunity, myopathy, post-infectious disorders

## Abstract

Juvenile dermatomyositis (JDM) is the most common inflammatory myopathy affecting children, being marked by chronic inflammation which mostly impacts on both skin and skeletal muscles; diagnostic criteria of JDM include an unforeseeable mixture of clinical features, while treatment modalities commonly require corticosteroids or immunosuppressant agents. Although the pathogenesis of JDM is not completely understood, several infectious triggers have been linked to its priming via anecdotal reports related to children. Pediatric cases of recent-onset JDM have been temporally associated to an infectious disease by the power of increased titers of circulating antibodies to a putative infectious agent, including parasites, and/or detectable viral RNA or bacterial DNA. With this narrative review we offer an update about JDM association with a host of infections, namely parvovirus B19, Epstein-Barr virus, Coxsackie virus, human immune deficiency virus, severe acute respiratory syndrome coronavirus 2, *Mycoplasma pneumoniae* and *Toxoplasma gondii*, as resulting from the medical literature. Few are the evidence-proved results addressing JDM as an unambiguous post-infectious disorder and available data specifically related to children are poor, highlighting the need of further research into the exploration between environmental cut-out factors and JDM.

## 1 Introduction

Juvenile dermatomyositis (JDM) is the most common inflammatory myopathy occurring in the pediatric age. Recent reviews have described the clinical, serological, instrumental and morphological features of JDM as well as its potential triggers engaged in the disease pathogenesis (1). More specifically, this myopathy is a chronic inflammatory disorder characterized by systemic capillary vasculopathy that primarily affects the skin and/or skeletal muscles, but also internal organs, as lungs, with prevalent distribution children aged 5-14 years: some patients may display typical features of JDM without skin involvement or without muscle involvement; however, both these tissues are largely affected with different clinical relevance in most cases, with potential involvement of further organs which might consistently influence the overall outcome and prognosis of patients (2).

In 2017 the European League Against Rheumatism and American College of Rheumatology developed the diagnostic criteria for juvenile inflammatory myopathies: a definite diagnosis in children (in the absence of a muscle biopsy) should require a total score ≥7.5, considering different scores emerging from patient’s age, weakness of the proximal upper extremities, weakness of the proximal lower extremities, neck flexors relatively weaker than neck extensors, leg proximal muscles relatively weaker than distal ones, presence of heliotrope rash, presence of the Gottron papules, i.e. erythematous-to-violaceous papules standing over the extensor surfaces of finger joints as well as elbows, knees, malleoli and toes, or the Gottron sign, i.e. erythematous-to-violaceous macules over the extensor surfaces of joints, presence of esophageal dysmotility, positivity of anti-Jo-1 (histidyl-tRNA synthetase) autoantibodies and elevated level of muscle enzymes, i.e. creatine kinase, lactate dehydrogenase, aspartate aminotransferase or aldolase (3). The positivity of anti-synthetase autoantibodies has been defined as a separate autoimmune condition characterized by distinct clinical features mostly including a lung-dominant disease resembling a severe-course idiopathic chronic interstitial pneumopathy (4). The cornerstone of JDM therapy at the time of diagnosis is based on high-dose corticosteroids in combination with methotrexate or other immunosuppressant agents (5), but less focused is its etiopathologic background.

The etiology of JDM has not been univocally determined, and - unlike the majority of other inflammatory rheumatologic diseases - JDM is rarely familial, suggesting a crucial role of environmental culprits, such as infectious agents (6). How a ubiquitous microrganism can promote an autoimmune disease in at-risk populations is not fully decoded. Indeed, according to different epidemiological studies, more than half of children who have developed JDM experienced respiratory symptoms like cough, sore throat or lower respiratory illness such as pneumonia, asthma and bronchitis before the clinical appearance of JDM (7). Furthermore, Tezak et al. showed a higher expression of many interferon (IFN)-induced genes in muscle biopsies from children with untreated naïve JDM than in controls, supporting the hypothesis of a frank immunologic response to theoretical infectious agents, aware that the transcription of IFN-inducible genes is a definite trademark of host’s defense mechanism against infections (8).

**Figure 1:**
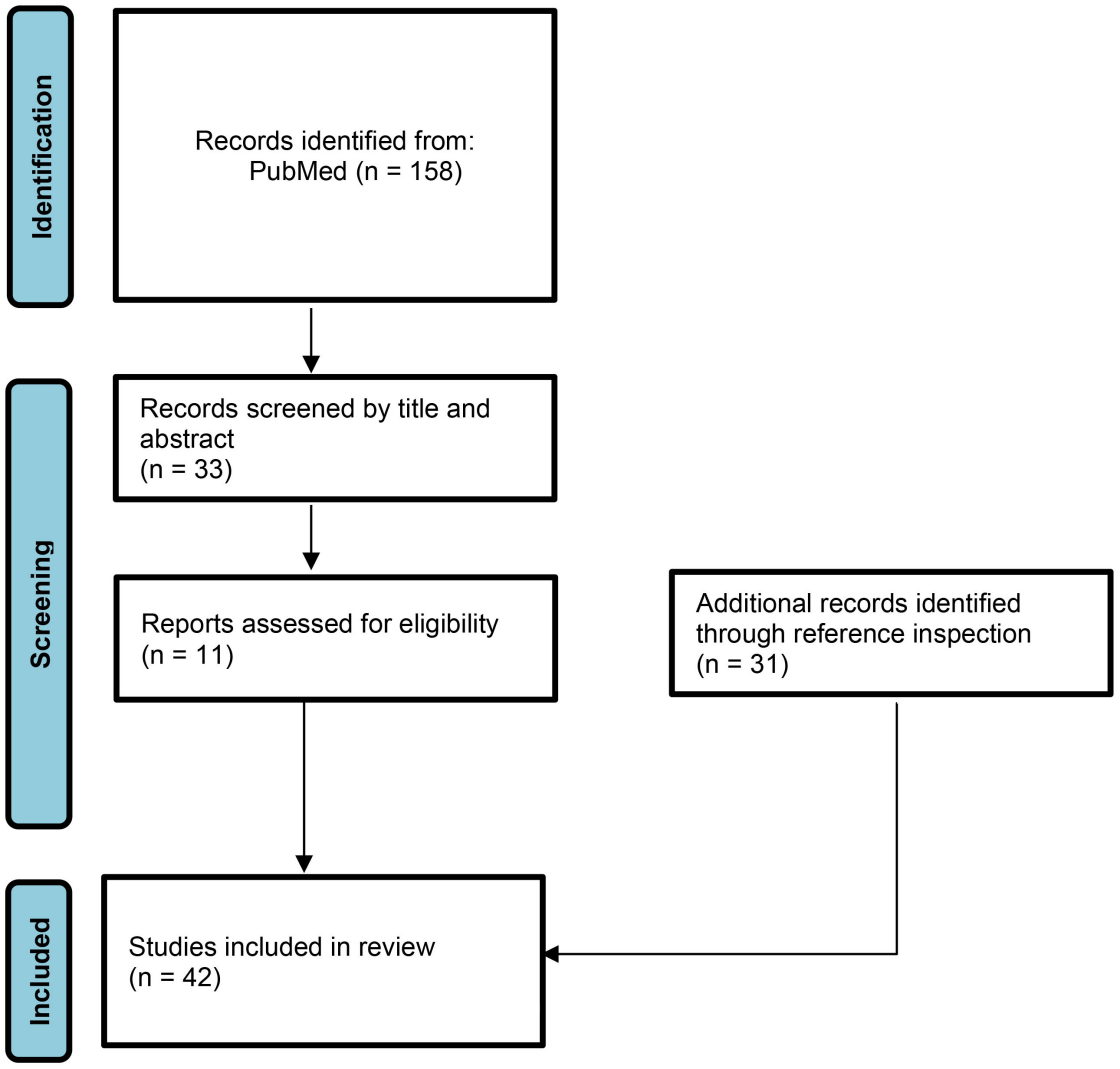
Literature’s research.

Therefore, in this review we focus on the potential links between infectious triggers and the onset of JDM according to the currently accumulated evidence and updated studies. A literature search was performed with a selection of papers dedicated to pediatric age, including randomized studies, systematic reviews and/or case reports associating a previous infection to onset of JDM. The literature search was carried out on the MEDLINE/PubMed database, with a choice of articles written in English published from 1970 to 2023 (**Figure 1**). Additionally, a manual search of bibliographies in the identified articles was also performed for the potential inclusion of other papers useful to keep updated the results about the review topic. The following combinations of keywords were used: “Juvenile dermatomyositis” AND “virus” OR “bacteria” OR “protozoan” OR “fungi” OR “infection” AND “children” OR “adolescent” OR “paediatric” OR “pediatric”. Agents identified in our literature search were parvovirus B19, Epstein-Barr virus, Coxsackie virus, human immunodeficiency virus, *Mycoplasma pneumoniae*, *Toxoplasma gondii*, and severe acute respiratory syndrome coronavirus 2.

## 2 Parvovirus B19

Chronic inflammation triggered by parvovirus B19-induced biosynthesis of the nonstructural NS1 protein and subsequent interleukin-6 overproduction may predispose parvovirus-infected patients to the development of autoimmune phenomena (9). Indeed, the NS1 protein promotes cell death by apoptosis in erythroid-lineage cells, and is also implicated in inducing the progression of different inflammatory pathways (10). Jalali et al. investigated the possible role of parvovirus B19 NS1 in the modulation of proinflammatory cytokines via transfection of human embryonic kidney cells, often referred to as HEK-293T cells, through a plasmid containing the fully sequenced *NS1* gene (11). Interleukin-6 mRNA was found increased in NS1-transfected cells, and cellular supernatants were collected to determine the type and quantity of cytokines produced. Overall, the authors found that proinflammatory cytokine levels were increased following the expression of human parvovirus B19 NS1 in the HEK-293T cells, suggesting the pivotal role of NS1 in the upregulation of inflammatory reactions (11). The hypothesis of a contribution by the parvovirus B19 in the onset of JDM was firstly promoted in 1995 as the parvovirus infection strictly preceded the symptomatic full-blown manifestations of JDM in one pediatric patient (12). Additionally, given the similarity of JDM presentation and parvovirus B19 infection, Mamyrova et al. performed a case-control study to determine if this infection could be associated with the occurrence of JDM compared to age-matched controls; plasma samples were obtained from 62 pediatric patients (their mean age was 9.7 years) with JDM, and all had been studied within 6 months since diagnosis (13). Concurrently, muscle biopsies were obtained before initiation of therapy from 4 patients with JDM, 2 of whom also provided plasma samples. The results of this investigation revealed that parvovirus B19 infection seemed not positively associated with JDM, and no increased prevalence of parvovirus B19 DNA in the muscle and plasma of patients with JDM was found in comparison with age-matched controls. In particular, the rate of parvovirus B19 seropositivity in patients with JDM was similar to that of healthy children (13). Nevertheless, it remains important to continue investigating the association between parvovirus infections and JDM onset or JDM flares in order to better understand the disease and to guide medical doctors and patients in best practices.

## 3 Epstein-Barr virus

Epstein-Barr virus (EBV) can induce a lifelong infection in a large number of infected patients due to in-depth disruption of normal immune control, and several autoimmune disorders have been associated with EBV since 1971, when different molecular mimicry mechanisms between systemic lupus erythematosus and specific EBV responses were associated with increased viral load, increased number of latently infected peripheral B cells, and impaired functional T cell activity (14). The underlying mechanism of EBV infection might be referred to impaired CD8+ T cell control and infiltration of autoreactive T cells into a target organ in genetically predisposed subjects, as proposed by Pender in 2012 (15). More specifically, a relationship with JDM was speculated in 2007 when Barzilai et al. tested a cohort of 1595 serum samples from 23 different autoimmune disease groups, finding significantly increased titers of anti-EBV antibodies in JDM patients compared to healthy subjects (16). More recently, higher titers of anti-EBV antibodies were found in newly-onset JDM patients, though with no significant statistical difference in comparison with controls: the authors found that CD3^-^CD16^+^CD56^+^ lymphocytes were extremely low in the earlier phases of JDM patients (17). Additional observational studies are undoubtedly needed to strengthen a potential correlation between EBV infection and onset of JDM.

## 4 Coxsackie virus

Coxsackie viruses can break immunological tolerance to self-antigens and have a role in the progression or exacerbation of different autoimmune disorders (18). This family of viruses has been included among factors that can contribute to both JDM pathogenesis and JDM flares in genetically susceptible subjects (19). Multiple studies have analyzed the serologic response to Coxsackie virus and viral RNA in muscle biopsies: the presence of antibodies against Coxsackie viruses in JDM patients’ serum was tested in two studies. In the first one, published in 1986, complement fixation-antibodies to Coxsackie B1, B2 and B4 viruses were significantly more frequently demonstrated in the serum of 12 JDM patients than in controls hospitalized for common viral infections (20). In the second one, published in 1997, there was no difference in frequency between the tested groups (21). Bowles et al. detected Coxsackie-related viral RNA in the muscle biopsy specimens of 4 out of 7 JDM patients as opposed to biopsy samples from 4 patients with Duchenne muscular dystrophy and 6 healthy controls (22). Pachman et al. used Coxsackie-B-virus-specific probes via reverse transcription of purified virus genomic RNA to test for the presence of virus RNA in muscle biopsy samples of 2 patients with adult dermatomyositis, 7 with JDM, 4 with Duchenne muscular dystrophy, and 6 normal controls: of the 9 patients with inflammatory muscle diseases, 5 were positive for Coxsackie B virus RNA, and no viral sequences were found in the other samples (23). Unfortunately, there are no updated articles investigating the link between Coxsackie virus or other enteroviruses and JDM onset, which remains a matter of debate.

## 5 Human immunodeficiency virus

Over the years it has been recognized that human immunodeficiency virus (HIV) can be associated with profound immunity disruption in patients with HIV infection (and subsequent acquired immune deficiency syndrome), in whom the setup of autoimmunity is intriguing. Yet, the spectrum of reported autoimmune phenomena in these patients is increasing. In the case of a frank loss of immunocompetence many autoimmune diseases that are predominantly T cell subtype CD8-driven can predominate in HIV patients, while B cell stimulation can lead to the production of different nonspecific autoantibodies, including anti-cardiolipin, anti-beta_2_ glycoprotein 1, anti-DNA, anti-small nuclear ribonucleoproteins, anti-thyroglobulin, anti-thyroid peroxidase, anti-erythropoietin, and anti-myosin antibodies (24). After a few descriptions in adults, only one pediatric case of an HIV-infected 8-year-old boy has been reported along with skin rashes, muscle weakness and polymicrobial infections following mild mmunosuppression: diagnosis of JDM was established by raised muscular enzymes, suggestive muscle imaging study, and muscle biopsy, while corticosteroids were successful (25). Carroll & Holmes emphasized that dermatomyositis may occur approximately 6-18 months after the detection of HIV infection, exhibiting an inverse relationship with the course of infection (26). Further studies have elucidated similarities and differences between anti-self responses in HIV infection and autoimmune diseases, identifying new molecular players that might enhance immune protection to HIV, and suggested that invasion by B and T lymphocytes into the perimysial and perifascicular spaces might lead to myocyte destruction, while dysfunctional B lymphocytes become prone to produce autoantibodies triggering immune complex formation and complement activation (27). Presumably, a concerted effect of multiple immune system failures sustained by HIV infection can trigger autoimmune phenomena, initiating and sustaining the pathogenesis of JDM.

## 6 Mycoplasma pneumoniae

An aberrant host immune response plays a critical role in the development of extra-pulmonary manifestations related to *Mycoplasma pneumoniae*, involving heart, skin, central nervous system, joints or muscles, even in the absence of a simple involvement of the lower respiratory tract (28). The pathomechanism of this satellite non-pulmonary involvement remains unknown, though it has been hypothesized that a proinflammatory cytokine-mediated organ damage with macrophage activation and vascular occlusion causing vasculitic or thrombotic complications might occur. A further factor which favors the *Mycoplasma pneumoniae*-associated non-pulmonary manifestations is the younger age, as they occur more often in children and younger adults (29). As a matter of fact, a 14-year-old girl with a 2-month history of progressive weakness and myalgia and a 2-week history of fever showed markedly increased creatinine kinase and substantially higher anti-*Mycoplasma pneumoniae* antibody response: clarithromycin was started and her myalgia abated, though a newly-onset heliotrope rash appeared (30). Hence, a myogenic pattern of polyphasic motor unit potentials was seen on the electromyogram, while muscle biopsy showed endomysial lymphocytic infiltration with muscle degeneration and chemochine-induced perivascular lymphocyte infiltration provoked by *Mycoplasma pneumoniae* infection (30). In addition, a 9-year-old girl with a 2-month history of generalized symmetrical muscle weakness and skin rashes was found to be previously infected by *Mycoplasma pneumoniae*, as revealed by high anti-*Mycoplasma pneumoniae* immunoglobulin M antibodies, with muscle biopsy revealing perifascicular atrophy, leading to diagnosis of JDM (31). The cascade of autoimmune reactions after *Mycoplasma pneumoniae* infection can directly or indirectly blight certain organs as muscles and give rise to JDM, but additional investigations are necessary to confirm its causative role.

## 7 Toxoplasma gondii

Some case reports highlighting the association between toxoplasmosis and dermatomyositis can be found in the medical literature. However, there are several problems in establishing the exact relationship of this parasite with JDM, mostly due to the widespread prevalence of anti-*Toxoplasma* antibodies in the general population. Most reported cases of JDM were due to the reactivation of a latent *Toxoplasma gondii* infection in immunocompromised hosts, as shown by a systematic review by Behan et al. (32). Pediatric cases having acute *Toxoplasma* infection were treated with specific antiprotozoal therapy and showed variable responses at the level of muscular manifestations. Some pediatric reports showed a clinical improvement after anti-parasitic drugs compared to immunosuppressive ones (33). Indeed, a 10-year-old girl with previous close contact with cats developed typical features of JDM, confirmed by muscle biopsy, while toxoplasmosis was demonstrated by the positive serologic test; only a minimal improvement could be observed after prednisone and azathioprine, while full improvement was obtained after 4 weeks of pyrimethamine and sulfadiazine (33). A further 13-year-old girl with JDM and concomitant toxoplasmosis did not improve under corticosteroids and immunosuppressive drugs, but only after anti-parasite therapy (34). Cuturic et al. proposed to consider two phases of the toxoplasmosis-related polymyositis: an acute one, responsive to antiprotozoal therapies, and a subsequent chronic one, requiring the administration of corticosteroids; they also suggested that early treatment against *Toxoplasma gondii* can be the most relevant variable affecting disease outcome, and that serological tests for toxoplasmosis should be a part of any initial evaluation for dermatomyositis (35).

## 8 Severe acute respiratory syndrome coronavirus 2

Severe acute respiratory syndrome coronavirus 2 (SARS-CoV-2) drives a marked inflammation with risk of cytokine storm (36): A very recent study has linked SARS-CoV-2 infection to the onset of different systemic autoimmune diseases, and the most common disorder found in adults was idiopathic inflammatory myopathy, with 4 cases of dermatomyositis, after an average time of 23.7 days from COVID-19 diagnosis (37). Holzer et al. reported 4 cases of new-onset dermatomyositis shortly after SARS-CoV-2 infection (for 1 case) and mRNA vaccination against SARS-CoV-2 (for 3 cases): all patients required intensive immunosuppressive treatment, and the authors concluded that SARS-CoV-2 infection and also mRNA COVID-19 vaccination may be regarded as possible triggers of the IFN-pathway, culminating with the production of dermatomyositis-specific autoantibodies like anti-melanoma differentiation-associated gene 5 (MDA5) and anti-nuclear matrix protein 2 (NXP2) antibodies, which are closely related to the defense against viruses (38). Anyway, due to the limited number of identified cases, this association requires further research for confirmation. Rodero et al. studied SARS-CoV-2 infection history in 10 children and adolescents with recent onset of JDM (6 cases) or with relapsed JDM (4 cases), seen in a single French center since the pandemic start, finding that 1 newly onset JDM and 1 relapsing patient had a very recent history of infection by SARS-CoV-2 (39). Their observation led to strongly suggest that SARS-CoV-2 infection could trigger the development of JDM, possibly through the induction of type I IFN-mediated response. Virus-induced muscle inflammation, varying in presentation from a typical dermatomyositis to diffuse rhabdomyolysis, could be attributed to the angiotensin-converting enzyme (ACE) receptor-mediated direct entry and to muscle fiber distress, switching on innate and adaptive immunity pathways (40). This theory is supported by the report of an 11-year-old girl with anti-MDA5-positive JDM and progressively severe interstitial lung disease, which had a fatal outcome due to SARS-CoV-2 infection and secondary hemophagocytic lymphohistiocytosis (41). Furthermore, a common pathogenesis of both COVID-19 and dermatomyositis might be explained by the fact that baricitinib, a Janus kinase inhibitor, has resulted effective in the treatment of severe COVID-19 patients, showing that COVID-19-related organ damage should at least partly be attributable to type I IFN activation, which is known to play a role in myofiber damage in the case of dermatomyositis (42). The exact mechanism of this partnership remains largely misunderstood, and further observations are needed.

## 9 Conclusions

JDM remains the most common inflammatory myopathy in childhood although its exact etiology is still far to be thoroughly elucidated. Apart from environmental and genetic factors, the model of an infection-triggered autoimmunity process has been often conjectured by clinicians and researchers in their real-life experience. **Table 1** summarizes the main studies on the relationship between infectious agents and juvenile dermatomyositis. Many infectious agents are suspected to induce autoimmunity or break the self-tolerance in genetically predisposed individuals, paving the road to immune-mediated phenomena which can be framed in the scenery of idiopathic autoimmune myopathies. Although a direct indisputable evidence for this association is still lacking for JDM, there are some reports and studies showing the potential contribution of protean pathogens to the pathogenesis of JDM. This review has listed the infectious agents which have been considered triggers of JDM, though few are the evidence-proved results addressing JDM as an unambiguous post-infectious disorder. Ongoing investigation into the connections between environmental risk factors and either onset or exacerbation of JDM is crucial to gain a deeper understanding of this disease.

**Table 1:** Main studies on the relationship between infectious agents and juvenile dermatomyositis.

Pathogen	Source	Study design	Laboratory tests	Results
Parvovirus B19	Mamyrova et al. (2005) (13)	Case-control study	Serology	No difference of Parvovirus B19 seropositivity prevalence between JDM patients and controls
			Search of DNA in muscle	No difference of Parvovirus B19 DNA prevalence in muscle between JDM patients and controls
			Search of DNA in plasma	No difference of Parvovirus B19 DNA prevalence in plasma between JDM patients and controls
Epstein-Barr virus	Barzilai et al. (2007) (16)	Case-control study	EBNA IgG, EBVCA IgM and IgG and EA IgG in serum	Increased EBV seropositivity prevalence in PM patients
	Zheng et al. (2021) (17)	Case-control study	EBV-CA IgG and EBNA IgG in serum	Increased EBV previous infection markers in JDM patients
			EBV-VCA IgM and EA IgG in serum	No difference of EBV acute infection markers prevalence between JDM patients and controls
			EBV DNA in plasma	None of the JDM patients or controls showed positive EBV-DNA in plasma
Coxsackie virus	Christensen et al. (1986) (20)	case-control study	complement fixation-antibodies to Coxsackie B1, B2 and B4 viruses	Increased Coxsackie seropositivity prevalence in JDM patients
	Bowles et al. (1987) (22)	case-control study	muscle biopsy	Increased Coxsackie RNA in the muscle biopsy specimens in JDM patients
	Pachman et al. (1997) (21)	case-control study	IgM antibodies	No difference of Coxsackie IgM prevalence between JDM patients and controls
	Pachman et al. (1995) (23)	case-control study	complement fixation-antibodies to Coxsackie virus	No difference of Coxsackie seropositivity prevalence between JDM patients and controls
			muscle biopsy	No difference of Coxsackie RNA in the muscle biopsy specimens between JDM patients and controls
Human immunodeficiency virus	Sharma et al. (2014) (25)	case report of an 8-years-old HIV infected boy	Skin and muscle biopsy	Evidence of features suggestive of dermatomyositis
*Mycoplasma pneumoniae*	Moon et al. (2015) (30)	Case report	Serology	Seropositivity for *Mycoplasma pneumoniae*
			Muscle biopsy	Endomysial lymphocytic infiltration with muscle degenerations/regenerations and perivascular lymphocyte infiltrations
	Anuar et al. (2021) (31)	Case report	Serology	Seropositivity for *Mycoplasma pneumoniae*
			Muscle biopsy	Perifascicular atrophy
*Toxoplasma gondii*	Schröter et al. (1987) (33)	Case report	Antibodies IgM, muscle biopsy	Seropositivity and clinical improvement after antiprotozoan therapy; aspecific biopsy
	Santos et al. (2009) (34)	Case report	Antibodies IgG, muscle biopsy	Elevated titer and clinical improvement after antiprotozoan therapy; aspecific biopsy
SARS-CoV-2	Kouranloo et al. (2023) (37)	Systematic review	JDM autoantibodies, SARS-CoV-2 PCR	SARS-CoV-2 positivity and JDM symptoms
	Holzer et al. (2022) (38)	Literature review	JDM autoantibodies, SARS-CoV-2 PCR	Increased SARS-CoV-2 seropositivity prevalence in JDM patients
	Rodero et al. (2022) (39)	Case report	JDM features, SARS-CoV-2 PCR	SARS-Cov2 infection followed by JDM symptoms
	Saud et al. (2021) (40)	Literature review	JDM autoantibodies, SARS-CoV-2 PCR	SARS-CoV-2 positivity and JDM symptoms
	Quintana et al. (2021) (41)	Case report	JDM features, SARS-CoV-2 PCR	No correlation between acute infection and JDM

EBV, Epstein-Barr virus; HIV, human immunodeficiency virus; JFM, juvenile dermatomyositis; PCR, polymerase chain reaction; SARS-CoV-2, severe acute respiratory syndrome coronavirus 2.

## Author contributions

CS: Investigation, Writing – original draft. CB: Investigation, Writing – original draft. MM: Data curation, Writing – review & editing. IT: Data curation, Writing – review & editing. DR: Conceptualization, Methodology, Supervision, Writing – original draft, Writing – review & editing. SE: Conceptualization, Funding acquisition, Resources, Supervision, Writing – original draft, Writing – review & editing.
